# Supplementary material for: Development of a computer-aided design software for the quantitative evaluation of aesthetic damage
Source: PLoS One. 2019 Dec 18;14(12):e0226322. doi: 10.1371/journal.pone.0226322 (PMC6919621; doi:10.1371/journal.pone.0226322)
Supplement: S1 Code site — 1. (ZIP) [file pone.0226322.s001.zip › [EESC_jr][Projeto]Relatorio_07_21/reports/templates_zip/gentelella-master/vendors/Flot/examples/tracking/index.html]

Flot Examples: Tracking


## Tracking

You can add crosshairs that'll track the mouse position, either on both axes or as here on only one.

If you combine it with listening on hover events, you can use it to track the intersection on the curves by interpolating the data points (look at the legend).

Copyright © 2007 - 2014 IOLA and Ole Laursen
